# Are distal radius fracture classifications reproducible? Intra and interobserver agreement

**DOI:** 10.1590/S1516-31802008000300008

**Published:** 2008-05-01

**Authors:** João Carlos Belloti, Marcel Jun Sugawara Tamaoki, Carlos Eduardo da Silveira Franciozi, João Baptista Gomes dos Santos, Daniel Balbachevsky, Eduardo Chap Chap, Walter Manna Albertoni, Flávio Faloppa

**Keywords:** Colles’ fracture, Radius fractures, Classification, Reproducibility of results, Validation studies, Fratura de Colles, Fraturas do rádio, Classificação, Reprodutibilidade dos testes, Estudos de validação

## Abstract

**CONTEXT AND OBJECTIVE:**

Various classification systems have been proposed for fractures of the distal radius, but the reliability of these classifications is seldom addressed. For a fracture classification to be useful, it must provide prognostic significance, interobserver reliability and intraobserver reproducibility. The aim here was to evaluate the intraobserver and interobserver agreement of distal radius fracture classifications.

**DESIGN AND SETTING:**

This was a validation study on interobserver and intraobserver reliability. It was developed in the Department of Orthopedics and Traumatology, Universidade Federal de São Paulo — Escola Paulista de Medicina.

**METHOD:**

X-rays from 98 cases of displaced distal radius fracture were evaluated by five observers: one third-year orthopedic resident (R3), one sixth-year undergraduate medical student (UG6), one radiologist physician (XRP), one orthopedic trauma specialist (OT) and one orthopedic hand surgery specialist (OHS). The radiographs were classified on three different occasions (times T1, T2 and T3) using the Universal (Cooney), Arbeitsgemeinschaft für Osteosynthesefragen/Association for the Study of Internal Fixation (AO/ASIF), Frykman and Fernández classifications. The kappa coefficient (κ) was applied to assess the degree of agreement.

**RESULTS:**

Among the three occasions, the highest mean intraobserver k was observed in the Universal classification (0.61), followed by Fernández (0.59), Frykman (0.55) and AO/ASIF (0.49). The interobserver agreement was unsatisfactory in all classifications. The Fernández classification showed the best agreement (0.44) and the worst was the Frykman classification (0.26).

**CONCLUSION:**

The low agreement levels observed in this study suggest that there is still no classification method with high reproducibility.

## RESUMO

**CONTEXTO E OBJETIVO:**

Para que as classificações das fraturas possam ser úteis, elas devem prover o prognóstico, apresentar concordância interobservador e reprodutibilidade intraobservador. O objetivo foi avaliar a concordância intra e interobservadores das classificações das fraturas do rádio distal.

**TIPO DE ESTUDO E LOCAL:**

Estudo de validação (concordância intra e interobservadores), desenvolvido no Departamento de Ortopedia e Traumatologia da Universidade Federal de São Paulo — Escola Paulista de Medicina (Unifesp-EPM), São Paulo, Brasil.

**MÉTODO:**

Foram avaliadas 90 fraturas do rádio distal com desvio por meio de radiografias por cinco observadores (um médico residente de Ortopedia do terceiro ano, um graduando do sexto ano de medicina, um médico radiologista, um ortopedista especializado em trauma e um ortopedista especializado em cirurgia da mão) em três momentos diferentes, empregando as classificações Universal (Cooney), AO/ASIF (Osteosynthesfragen/Association for the Study of Internal Fixation), Frykman e Fernández. Aplicou-se o coeficiente de concordância kappa (κ) para avaliação das classificações.

**RESULTADOS:**

O maior κ intraobservador médio, se considerarmos os três momentos, foi da classificação Universal (κ = 0,61), seguida da Fernández (κ = 0,59), Frykman (κ = 0,55) e AO/ASIF (κ = 0,49). A concordância interobservador foi insatisfatória em todas as classificações. A classificação de Fernández mostrou a melhor concordância (κ = 0,44) e a pior foi a de Frykman (κ = 0,26).

**CONCLUSÃO:**

Os baixos níveis de concordância observados neste estudo sugerem que atualmente ainda não há um método de classificação plenamente reprodutível.

## INTRODUCTION

Distal radius fractures have an approximate incidence of 1:10,000 people and represent 16% of skeletal and 74% of forearm fractures.^1^ They are more prevalent among females and present a progressive increase in complications with age, as osteopenia and osteoporosis become more prevalent.^2^ The most common trauma mechanism is falling over onto the hand.^3^ The characteristics of such fractures (trace location, possible joint involvement, comminution and degree of soft-part lesion) are directly related to the force of the trauma, wrist angle at the moment of the trauma and bone health.^2^

Systems have been developed to help surgeons in classifying fractures into different and clinically useful groups for treatment definition. Colles, Smith, Pouteau, and others described fracture morphology with a view to treatment.^3-5^ With the advent of radiology, it became possible to describe injuries more precisely, including both the degree of displacement and the presence of joint injuries. In 1951, Garland and Werley^6^ created a classification based on the presence or absence of joint involvement, metaphyseal comminution and/or angular deformity. In 1959, Lindstrom expanded these criteria to six groups, describing the fragment displacement in further detail, along with joint involvement.^7^

In 1967, Frykman established a rating system that considered the radiocarpal and/or distal radius-ulna joints, and also the presence or absence of the ulnar styloid.^8^ Even thus, this was a limited rating system: it did not consider factors like the extent of fragment displacement, presence or absence of comminution and instability factors.

In 1984, Melone^9^ published a rating system for distal radius joint fractures based on four parts: radius styloid, radius shaft, dorsal fragment and palmar radius. This rating system has been used to define surgical fixation methods, but its accuracy and reproducibility for identifying the four fragments on conventional x-rays have not been validated yet by clinical trials, and the system still presents disagreements.^10^

The Arbeitsgemeinschaft für Osteosynthesefragen/Association for the Study of Internal Fixation (AO/ASIF) rating system was created in 1986 and reviewed in 1990. It considers bone injury severity and is a basis for treatment and results evaluation. There are three basic lesion types in this system: extra-articular, partial articular and complete articular. The three groups are organized into increasing order of severity of morphological complexity, treatment difficulty and prognostics. It is one of the most complete ratings available, but its intra and interobserver reproducibility has been a problem when evaluating groups and subgroups.^11,12^

The Universal rating system described by Cooney^13^ is characterized by simplicity, classifying fractures as intra or extra-articular, displacement present or absent, and according to the degree of stability and possibilities of reduction. It thus acts as a guide for treatment patterns.

The rating system proposed by Fernández is based on the trauma mechanism.^14^ This rating was created to be practical, predict stability, check on associated fractures of the ulna styloid process, identify equivalent lesions in children and make general recommendations for treatment.

To be considered good, a rating system must be valid, reliable and reproducible. Furthermore, an ideal rating system should standardize a trustworthy communication language that provides guidelines for treatment, indicates the possibilities of complications, evaluates fracture stability and enables fracture prognosis. This ideal system should also provide a mechanism that allows comparison of the results obtained with treatments undertaken on similar fractures in other centers, reported at different times in the literature.^15^

Variation in evaluators’ expertise may have influenced evaluations carried out on intraobserver and interobserver agreement. Studies have shown that less experienced observers attain lower rates of intraobserver agreement than do expert physicians.^12,16^ However, in a comparison of one group in which the observers were more experienced in rating assessments with another group whose expertise was lower, no significant difference in interobserver agreement was found.^12^ It would also be expected that, as observers study and become accustomed to using a given rating system, the agreement between them, and within their own observations, would increase. Yet, it was observed that repeated application, i.e. at different moments in time, of the same rating system, had no impact on intraobserver and interobserver reproducibility.^10^

Considering the high prevalence of these kinds of fracture and the need to properly and reproducibly classify them, we developed the present study. Its aim was to evaluate the reproducibility of the four most widely used rating systems in our field.^16^

## OBJECTIVE

This objective of this study was to evaluate the intraobserver and interobserver agreement of the Universal, AO/ASIF, Frykman and Fernández rating systems for fractures with regard to displacement of the radius distal extremity.

## MATERIAL AND METHODS

This was a ratings reproducibility study using the kappa index. Ninety-eight displaced distal radius fractures in 96 patients over the age of 40 years who had been treated at the Hand Institute of Universidade Federal de São Paulo — Escola Paulista de Medicina (Unifesp-EPM) were retrospectively evaluated from the radiographic archives. Five observers were involved: one third-year orthopedic resident (R3), one sixth-year undergraduate medical student (UG6), one radiologist physician (XRP), one orthopedic trauma specialist (OT) and one orthopedic hand surgery specialist (OHS). These observers used four classification systems to label each case using simple x-rays in two incidence planes (posteroanterior and lateral to the wrist). The classifications used were the Universal (Cooney), AO/ASIF, Frykman and Fernández, and these were previously presented and explained to the evaluators, with an illustrated brochure showing descriptions of degrees and types of injury.

At the first evaluation (time T1), all the x-rays were assessed in numerical sequence. Three weeks later, at the second evaluation (time T2), the initial x-ray order was randomly changed to generate a new sequence. A further randomization of the sequence was performed for the third evaluation (time T3), after six weeks. The x-rays were scanned and analyzed in computers. Data were collected on spreadsheets and the kappa (κ) coefficient was used to assess agreements.

k was applied using the method proposed by Fleiss et al.^17^, and the random expected agreement calculation described by Scott^18^ and Cohen^19^ was also used. The latter two methods enable calculation of agreements for multiple (more than two) observers with regard to evaluations of nominal variances. They have therefore frequently been used in studies to evaluate intraobserver and interobserver reliability and reproducibility. The kappa agreement coefficient provides a parallel rating of the agreement among the observers that is randomly correct. Kappa values range from -1 to +1; values between -1 and 0 indicate that the observed agreement was lower than what was randomly expected, 0 indicates the random agreement level, and +1 indicates total agreement.^17^ In general, kappa values of less than 0.5 are considered unsatisfactory; values between 0.5 and 0.75 are considered satisfactory and appropriate, and values above 0.75 are considered excellent.^20^

This project was approved by the Research Ethics Committee of Unifesp-EPM, under No. 1076-06, on August 4, 2006.

## RESULTS

Out of the initial 98 fractures, eight were excluded: four presented poor quality x-rays and another four presented x-rays produced with the forearm immobilized in plaster. Thus, the sample size was reduced to 90 fractures.

The highest mean intraobserver κ, taking all three observation times, was from the Universal classification (κ = 0.61), followed by Fernández (κ = 0.59), Frykman (κ = 0.55) and AO/ASIF (κ = 0.49) (Table 1).

**Table 1:** Intraobserver kappa values between the three times (T1, T2 and T3)

Observer	Classification			
	Universal	AO/ASIF	Frykman	Fernández
OHS	0.6568	0.6362	0.6375	0.7115
OT	0.6452	0.634	0.6632	0.5274
XRP	0.3513	0.3111	0.3099	0.2882
R3	0.7589	0.4835	0.549	0.7412
UG6	0.6406	0.3751	0.5829	0.6812
**Mean kappa**	**0.61056**	**0.48798**	**0.5485**	**0.5899**

*R3 = third-year orthopedic resident; UG6 = sixth-year medical student; XRP = radiologist physician; OHS = orthopedic hand surgery specialist; OT = orthopedic trauma specialist; AO/ASIF = Arbeitsgemeinschaft für Osteosynthesefragen/Association for the Study of Internal Fixation.*

Evaluation of the intraobserver k between the times T1 and T2 showed that the highest mean was from the Fernández classification (κ = 0.58), followed by the Universal (κ = 0.56), and the lowest mean was from the AO/ASIF (κ = 0.46) (Table 2).

**Table 2:** Intraobserver kappa values between times T1 and T2

Observer	Classification			
	Universal	AO/ASIF	Frykman	Fernández
OHS	0.6914	0.6284	0.6121	0.738
OT	0.5784	0.623	0.6075	0.4933
XRP	0.2478	0.2144	0.2971	0.2638
R3	0.7089	0.4341	0.6076	0.7699
UG6	0.5821	0.385	0.5036	0.626
**Mean kappa**	**0.56172**	**0.45698**	**0.52558**	**0.5782**

*R3 = third-year orthopedic resident; UG6 = sixth-year medical student; XRP = radiologist physician; OHS = orthopedic hand surgery specialist; OT = orthopedic trauma specialist; AO/ASIF = Arbeitsgemeinschaft für Osteosynthesefragen/Association for the Study of Internal Fixation.*

Between times T2 and T3, the mean intraobserver κ was greater, ranging from κ = 0.59 for the Frykman classification to κ = 0.67 for the Universal classification (Table 3).

**Table 3:** Intraobserver kappa values between times T2 and T3

Observer	Classification			
	Universal	AO/ASIF	Frykman	Fernández
OHS	0.6597	0.7076	0.6896	0.7721
OT	0.6905	0.6618	0.7112	0.4433
XRP	0.4523	0.4381	0.3116	0.3775
R3	0.8909	0.6927	0.5397	0.8023
UG6	0.6615	0.5504	0.7024	0.8117
**Mean kappa**	**0.67098**	**0.61012**	**0.5909**	**0.64138**

*R3 = third-year orthopedic resident; UG6 = sixth-year medical student; XRP = radiologist physician; OHS = orthopedic hand surgery specialist; OT = orthopedic trauma specialist; AO/ASIF = Arbeitsgemeinschaft für Osteosynthesefragen/Association for the Study of Internal Fixation.*

All the mean interobserver k values for the classifications were higher at time 3, such that they were (in decreasing order) Fernández κ = 0.44, Universal κ = 0.41, AO/ASIF κ = 0.31 and Frykman κ = 0.26 (Table 4).

**Table 4:** Interobserver kappa values at each time (T1, T2, T3)

Time	Classification			
	Universal	AO/ASIF	Frykman	Fernández
T1	0.3963	0.2702	0.2427	0.34
T2	0.4004	0.2988	0.2589	0.4087
T3	0.4118	0.3117	0.2608	0.4344

*AO/ASIF = Arbeitsgemeinschaft für Osteosynthesefragen/ Association for the Study of Internal Fixation.*

Evaluation of the interobserver k by comparing pairs of observers at time 1 showed that the highest agreement was between the observers R3 and UG6 (0.60) in the Fernández classification. On the other hand, the lowest agreement was between XRP and UG6 (0.06), in the same classification system (Table 5).

**Table 5:** Analysis of interobserver kappa values for pairs at time 1 (T1)

Pairs	Classification			
	Universal	AO/ASIF	Frykman	Fernández
OHS versus OT	0.5429	0.4911	0.4007	0.3654
OHS versus XRP	0.2097	0.1635	0.1733	0.3036
OHS versus R3	0.5465	0.4532	0.2999	0.6849
OHS versus UG6	0.3683	0.1644	0.0954	0.4304
OT versus XRP	0.3463	0.2158	0.2468	0.2186
OT versus R3	0.594	0.4272	0.4173	0.3505
OT versus UG6	0.4453	0.2431	0.2225	0.3188
XRP versus R3	0.2689	0.1497	0.2513	0.1548
XRP versus UG6	0.1958	0.2158	0.1686	0.0584
R3 versus UG6	0.5726	0.2872	0.24	0.6029

*R3 = third-year orthopedic resident; UG6 = sixth-year medical student; XRP = radiologist physician; OHS = orthopedic hand surgery specialist; OT = orthopedic trauma specialist; AO/ASIF = Arbeitsgemeinschaft für Osteosynthesefragen/Association for the Study of Internal Fixation.*

At time 2, the highest agreement was obtained between OHS and R3 (0.77) in the Fernández classification, while the lowest was between XRP and R3 (0.12) in the AO/ASIF system (Table 6).

**Table 6:** Analysis of interobserver kappa values for pairs at time 2 (T2)

Pairs	Classification			
	Universal	AO/ASIF	Frykman	Fernández
OHS versus OT	0.5751	0.4889	0.399	0.4102
OHS versus XRP	0.2338	0.2191	0.1373	0.3286
OHS versus R3	0.4934	0.4602	0.308	0.7691
OHS versus UG6	0.556	0.3695	0.1495	0.4989
OT versus XRP	0.2769	0.1808	0.2444	0.2737
OT versus R3	0.5285	0.5285	0.4117	0.4193
OT versus UG6	0.5428	0.3342	0.2949	0.4153
XRP versus R3	0.2112	0.1174	0.2225	0.3397
XRP versus UG6	0.2187	0.1272	0.2748	0.205
R3 versus UG6	0.4368	0.3646	0.2422	0.4926

*R3 = third-year orthopedic resident; UG6 = sixth-year medical student; XRP = radiologist physician; OHS = orthopedic hand surgery specialist; OT = orthopedic trauma specialist; AO/ASIF = Arbeitsgemeinschaft für Osteosynthesefragen/Association for the Study of Internal Fixation.*

At time 3, the highest κ was between OHS and R3 (0.6) in the Fernández classification, while the lowest was between XRP and UG6 (0.1) in the AO/ASIF system (Table 7).

**Table 7:** Analysis of interobserver kappa values for pairs at time 3 (T3)

Pairs	Classification			
	Universal	AO/ASIF	Frykman	Fernández
OHS versus OT	0.5441	0.5382	0.4512	0.4781
OHS versus XRP	0.2692	0.2175	0.297	0.3492
OHS versus R3	0.532	0.4985	0.3277	0.6471
OHS versus UG6	0.5011	0.4618	0.1265	0.5337
OT versus XRP	0.3481	0.222	0.4361	0.293
OT versus R3	0.5491	0.4306	0.2638	0.4564
OT versus UG6	0.5142	0.3328	0.2312	0.4769
XRP versus R3	0.2471	0.1551	0.1759	0.3381
XRP versus UG6	0.2244	0.1005	0.2337	0.3505
R3 versus UG6	0.5118	0.4143	0.2105	0.534

*R3 = third-year orthopedic resident; UG6 = sixth-year medical student; XRP = radiologist physician; OHS = orthopedic hand surgery specialist; OT = orthopedic trauma specialist; AO/ASIF = Arbeitsgemeinschaft für Osteosynthesefragen/Association for the Study of Internal Fixation.*

## DISCUSSION

The four classification systems evaluated in the present study were chosen because they are the ones that are most widely studied and used in our field to classify distal radius fractures.^21^

In the Frykman classification, the general mean kappa value for intraobserver agreement was satisfactory (0.55), although the radiologist physician (XRP) presented an unsatisfactory value (0.31) that was far from the other four observers. After recalculating the intraobserver kappa without the medical student (UG6) and the orthopedic resident (R3), who were less experienced evaluators, the kappa value decreased to 0.54. This showed that the professional’s expertise level had no significant impact on the intraobserver agreement. Variance analysis between the observation times showed that UG6 presented relatively high variance (0.51 to 0.70) that was 39% greater than among the other observers. This probably resulted from the learning process required to become accustomed to this classification system. This assumption is reinforced by the observation that there was relatively lower variance among the more experienced observers at the same times. This suggests that the observer’s conditioning and knowledge, specific to the Frykman system, had a significant impact on the reproducibility obtained. It is important to make it clear that the professional expertise level was different from the level of experience relating to the classification. The k-value for the intraobserver agreement in the Frykman classification evaluated by Andersen et al.^10^ in 1996 was 0.48. In 1998, Illarramendi et al.^22^ in 1998 found κ = 0.61, and in 2003, Oliveira Filho et al.^16^ found κ = 0.55. These coefficients reported in literature were in line with the results from the present study (κ = 0.55).

With regard to the observer’s experience, the study published by Oliveira Filho et al.^16^ had similar conclusions to ours, thus demonstrating the positive effect of expertise on the agreement rate.

The interobserver agreement rate for the Frykman classification was unsatisfactory, albeit with a progressive increase from T1 (0.2427) to T3 (0.2608). However, this increase was relatively lower than what was observed from the other classification systems.

The analysis showed that, in comparison with the most experienced observers (OHS and OT), the XRP observer presented lower agreement rates. This suggests that although the XRP observer had professional experience with radiographic evaluations, this observer was not using these classification methods routinely. This demonstrates that professional experience of radiographic evaluation is not, on its own, a determining factor for a higher agreement rate using these classification systems. We also saw this when analyzing the other classification methods.

In our study, the interobserver reproducibility of the Frykman classification was unsatisfactory (0.26 at T3), and the k value was relatively lower than found in the studies by Andersen et al.^10^ and Illarramendi et al.^22^, which presented k of 0.35 and 0.43 respectively. Our unsatisfactory result from the Frykman classification probably results from the low agreement rate between XRP and the other evaluators.

The Universal classification evaluates the following variables of distal radius fractures, exclusively based on radiographic criteria: involvement or non-involvement of the radiocarpal joint, presence or absence of dislocation, fracture reducibility and stability. The biggest difficulty found in applying this classification was in assessing the degree of instability of the fracture. The literature did not demonstrate any consensus regarding the best way to predict specific instability criteria on the initial x-ray, and there are several studies with discordant results concerning such criteria.^23-26^

In the Universal classification, the average intraobserver index was satisfactory (0.61056). When the intraobserver kappa was recalculated without the less experienced observers (R3 and UG6), there was a reduction in kappa to 0.5511. This demonstrated that the degree of expertise did not influence the results, since an increased kappa would be expected when excluding the less experienced evaluators. On the other hand, analysis of how the agreement evolved from time T1 to M3 showed that UG6 presented an increase of 13%, which was lower than what was observed for R3 (increase of 25.7%) and XRP (increase of 82.5%). The intraobserver agreement for the Universal classification was also satisfactory in another study,^16^ which found κ = 0.54. However, that study demonstrated that the observer’s experience was a factor that modified the agreement.

The interobserver agreement for the Universal classification was unsatisfactory, but presented a progressive increase from T1 (0.3963) to T3 (0.4118). The XRP evaluator presented a lower agreement rate than what would be expected. However, we found that this observer’s agreement rate increased in relation to the OT and OHS evaluators. This suggests that conditioning to the Universal classification (i.e. the evolution from T1 to T3) was a factor that acted positively on the reproducibility.

The same difficulty described above for the Universal classification was found in the AO/ASIF application, even considering that in the latter, evaluation of the comminution location is extremely important for defining the groups.^27^ It is possible that this difficulty is the limiting factor for unsatisfactory agreement rates that have been found in previous studies.^10,16^

Assuming that the presence and location of comminution are determining variables with regard to fracture stability, thereby definitively guiding the therapy, detailed investigation of the reproducibility of these variables on the radiograph becomes necessary.

In the AO/ASIF classification, we used groups and subgroups (nine types) and the mean intraobserver value was unsatisfactory (0.49). There was a significant difference between the values for the more experienced observers (OHS κ = 0.64 and OT κ = 0.64) and those for the less experienced ones (R3 κ = 0.4835 and UG6 κ = 0.3751). This suggests that the expertise level had an influence. Only the XRP observer presented a value at odds with what was expected (κ = 0.34) for the more experienced evaluators. When the intraobserver kappa was recalculated without the less experienced observers (R3 and UG6), there was an increase in κ to 0.53, which reinforces the hypothesis that the professional expertise level had a significant impact on the intraobserver agreement. The analysis of variation between the times T1 and T3 demonstrated that the UG6 observer (less experienced) had an increase in agreement of 43%, XRP increased by 59.6%) and R3 increased by 104.3%. This demonstrated that conditioning to the classification had a significant impact on intraobserver reproducibility, particularly among the individuals with less expertise in using it.

In the literature, we saw that κ ranged from 0.37 to 0.60 in different studies,^10,12,16,22^ thus suggesting that the intraobserver reproducibility of AO/ASIF should be close to 0.5. In the present study, the mean κ was 0.48, with a range from 0.31 to 0.63. It was only in the study by Andersen et al.^10^, that the professional expertise level had no significant impact on intraobserver reproducibility. This could be explained by the presence of radiologist and orthopedist observers who were working in similar fields and frequently applied the AO/ASIF classification, in the same way as in our study. In the other studies,^12,16,22^ expertise played a modifying role in relation to intraobserver reproducibility.

The interobserver agreement for the AO/ASIF classification was also unsatisfactory, but presented progressive increase from T1 (0.27) to T3 (0.31). The XRP evaluator presented increased agreement with OT and OHS, by 0.8% and 3.0% respectively, and the UG6 observer presented increased agreement with OT and OHS, by 36.9% and 180.9%. This suggests that conditioning was a factor acting positively towards interobserver reproducibility, particularly for the less experienced individuals.

In the literature,^10,12,16,22^ we saw that the interobserver κ ranged from 0.3 to 0.5. This suggests that the AO/ASIF kappa is close to 0.4, implying unsatisfactory reproducibility. It also suggests that the professional expertise level had no impact on interobserver reproducibility in this classification, in the same way as seen in our study.

In the Fernández classification, the mean intraobserver κ was satisfactory (κ = 0.59). When the intraobserver kappa was recalculated without the less experienced observers, there was a reduction in κ (0.51), thus demonstrating that professional expertise did not have any influence on intraobserver agreement. Likewise, professional experience was not seen to have any positive influence on interobserver agreement between the times T1 and T3.

Conditioning (through evolution from T1 to T3) was seen to be a factor acting positively on intraobserver reproducibility, for the Fernández classification. There are no equivalent studies on this classification in the literature, which makes it impossible to make comparisons with the present results.

Regarding the interobserver agreement for this classification, it could be seen that there was a progressive increase in agreement between T1 (κ = 0.34) and T3 (κ = 0.44). This was relatively greater than what was seen in the other classifications. This suggests that the conditioning in this classification had a greater impact on reproducibility than did the conditioning in other classifications.

It is important to mention that the present study was limited to evaluating the agreement between the observers’ opinions. The study was unable to measure the accuracy of each observer’s opinion. To clarify the accuracy issue, studies in which clinical-radiographic diagnoses made by each observer were compared with an examination result or a standard procedure, i.e. one with high sensitivity and specificity, would be needed in order to prove the proposed diagnosis.

## CONCLUSIONS

The agreement rates observed in the present study show that currently there is still no classification method that is fully reproducible.

The best interobserver reproducibility rate was observed in the Fernández classification (0.43) and the worst was in the Frykman classification (0.26). The intraobserver reproducibility was satisfactory in the Universal (0.61), Fernández (0.59) and Frykman (0.55) classifications, and it was unsatisfactory in the AO/ASIF classification (0.49).

### Implications for further research

There is a need to perform new studies aimed at clarifying which classification variables present the highest disagreement rates between observers, with consequent limits to reproducibility. In the continuing search for an ideal classification, prospective studies to describe which variables can predict the instability factors in such fractures through radiographic examination are necessary.
